# Systemic signaling during abiotic stress combination in plants

**DOI:** 10.1073/pnas.2005077117

**Published:** 2020-05-29

**Authors:** Sara I. Zandalinas, Yosef Fichman, Amith R. Devireddy, Soham Sengupta, Rajeev K. Azad, Ron Mittler

**Affiliations:** ^a^Division of Plant Sciences, College of Agriculture, Food and Natural Resources, Christopher S. Bond Life Sciences Center, University of Missouri, Columbia, MO 65201;; ^b^Interdisciplinary Plant Group, Christopher S. Bond Life Sciences Center, University of Missouri, Columbia, MO 65201;; ^c^Department of Biological Sciences, College of Science, University of North Texas, Denton, TX 76203-5017;; ^d^Department of Mathematics, College of Science, University of North Texas, Denton, TX 76203-5017;; ^e^The Department of Surgery, University of Missouri School of Medicine, Christopher S. Bond Life Sciences Center, University of Missouri, Columbia, MO 65201

**Keywords:** abiotic stress, reactive oxygen species, stress combination, systemic acquired acclimation, systemic signaling

## Abstract

Extreme environmental conditions, such as heat, salinity, and decreased water availability, can have a devastating impact on plant growth and productivity, potentially resulting in the collapse of entire ecosystems. Stress-induced systemic signaling and systemic acquired acclimation play canonical roles in plant survival during episodes of environmental stress. Recent studies revealed that in response to a single abiotic stress, applied to a single leaf, plants mount a comprehensive stress-specific systemic response that includes the accumulation of many different stress-specific transcripts and metabolites, as well as a coordinated stress-specific whole-plant stomatal response. However, in nature plants are routinely subjected to a combination of two or more different abiotic stresses, each potentially triggering its own stress-specific systemic response, highlighting a new fundamental question in plant biology: are plants capable of integrating two different systemic signals simultaneously generated during conditions of stress combination? Here we show that plants can integrate two different systemic signals simultaneously generated during stress combination, and that the manner in which plants sense the different stresses that trigger these signals (i.e., at the same or different parts of the plant) makes a significant difference in how fast and efficient they induce systemic reactive oxygen species (ROS) signals; transcriptomic, hormonal, and stomatal responses; as well as plant acclimation. Our results shed light on how plants acclimate to their environment and survive a combination of different abiotic stresses. In addition, they highlight a key role for systemic ROS signals in coordinating the response of different leaves to stress.

## Significance

Environmental stresses such as heat, drought, and salinity, especially in combination with intense light conditions, can have devastating economical and sociological impacts. Although our knowledge of how each of these stresses affects plants when applied individually is vast, we know very little about how plants acclimate to a combination of different stresses. Here we reveal that plants can integrate different local and systemic signals generated during conditions of stress combination. We further show that the specific part at which plants sense the two co-occurring stresses makes a significant difference in how fast and efficient they acclimate. Our results shed light on how plants acclimate to and survive a combination of different stresses.

Abiotic stress conditions, such as heat, salinity, and decreased water availability, can have a devastating impact on plant growth and productivity, potentially resulting in extensive yield losses to agriculture, as well as the collapse of entire ecosystems (1, 2). To withstand harsh environmental conditions, plants evolved sophisticated perception, signaling, and acclimation mechanisms that allow them to survive different stress conditions, even at the cost of reduced growth and yield (2). However, successful acclimation of plants to stress conditions requires an efficient, timely, and coordinated response that spans most, if not all, parts and tissues of the plant (3). To achieve such as a coordinated response, plants evolved multiple systemic signaling pathways that allow them to communicate different stress signals from a particular part of the plant, that initially sensed the stress (i.e., local tissue), to the entire plant (i.e., systemic tissue), within minutes (3–15). Once these systemic signals are perceived in the systemic tissues of plants, they induce an acclimation process, termed “systemic acquired acclimation” (SAA) (16), that enables these tissues to withstand the stress even if they did not sense or experience it yet (5, 6, 15).

Among the different systemic signals found to propagate from a stressed local tissue to the entire plant within minutes are electric, calcium, reactive oxygen species (ROS), and hydraulic waves (3–15, 17), as well as changes in the levels of the plant hormones jasmonic acid (JA), abscisic acid, ethylene, and different metabolites (18). Rapid and systemic whole-plant transmission of electric, calcium, and ROS waves was recently shown to be required for plant acclimation to heat or light stresses, as well as for systemic wound responses (4–7, 15). Although these systemic signals were demonstrated to function in response to a single stress stimuli affecting a particular leaf or a root tip of the plant (4–7, 15), in nature plants frequently encounter more than one environmental stress condition at a time, resulting in a condition termed “stress combination” (19–22). Acclimation to a state of stress combination (e.g., a combination of drought and heat) has been shown to involve integrating responses to each of the individual stresses that simultaneously impact the plant (e.g., drought or heat), as well as the induction of a new type of response, sometimes involving thousands of transcripts, that is unique to the state of stress combination (19–22).

The different abiotic stresses that simultaneously impact a plant during stress combination may be sensed by the same or different parts (or tissues) of the plant (3). In addition, it was found that each different abiotic stress sensed by the plant (e.g., wounding, high light or heat stress) will trigger its own abiotic stress-specific systemic signaling and acclimation responses that include the accumulation of many different stress-specific transcripts and metabolites, as well as a coordinated stress-specific canopy-wide stomatal response (5, 23). The possible coactivation of different systemic signals in the same plant during stress combination raises a new fundamental question in plant biology: are plants capable of integrating different systemic signals that simultaneously originate at the same or different parts of the plant during stress combination?

To address this question, we studied the local and systemic response of the flowering plant *Arabidopsis thaliana* to a combination heat and light stress applied to the same or two different leaves of the same plant.

## Results

### Systemic Response of Plants to a Combination of High Light and Heat Stress Applied to a Single Leaf.

**Fig. 1.**
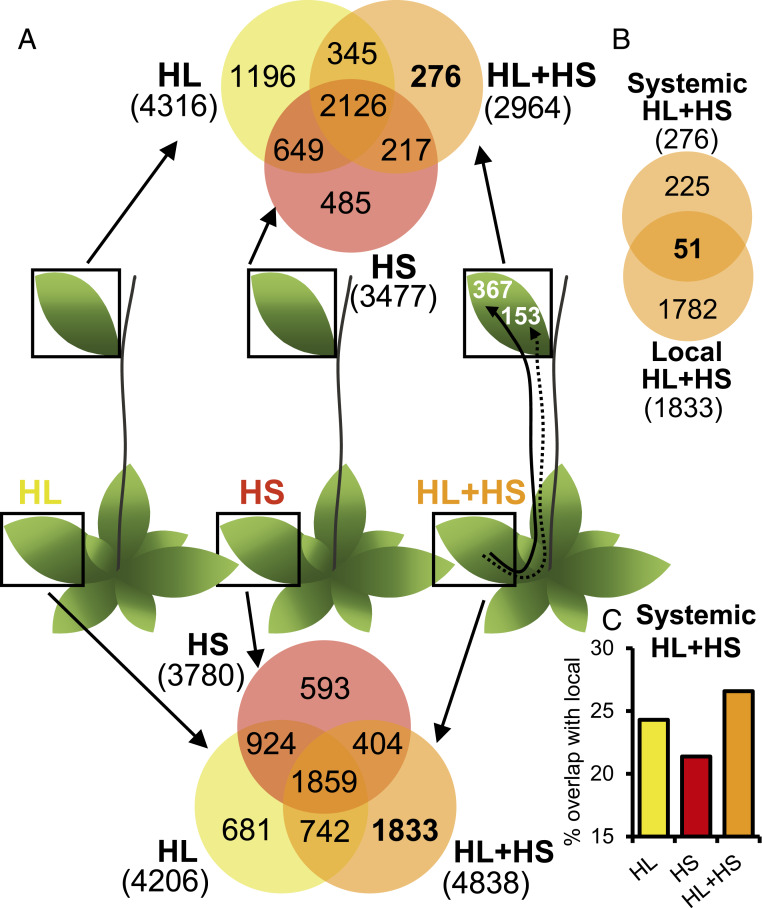
Plants can integrate two different systemic signals generated simultaneously at the same leaf during stress combination. (*A*) Overlap between the transcriptomic response of plants to a local treatment of HL, HS, or a combination of heat and light stresses applied to the same leaf (HL+HS). Venn diagrams for the overlap between the different responses are shown at the bottom for local leaves and at the top for systemic leaves. Black arrows in HL+HS plants represent the number of HL-specific (solid) or HS-specific (dashed) transcripts common between local and systemic leaves. (*B*) Venn diagram showing the overlap between stress combination-specific transcripts in local and systemic leaves. (*C*) Bar graph showing the percent overlap between the systemic HL+HS response and local HL, HS, or HL+HS responses. Local leaves were subjected to HL, HS, or a combination of HL+HS, and local and systemic leaves were sampled at 2 and 8 min following stress application. All experiments were repeated at least three times with 40 plants per biological repeat. All transcripts shown were significantly different from controls at *P* < 0.05 (negative binomial Wald test followed by Benjamini–Hochberg correction).

To study systemic signal integration during stress combination, we subjected a single leaf of *Arabidopsis* to a local treatment of high light (HL), heat stress (HS), or a combination of light and heat stresses (applied to the same leaf), and studied local and systemic responses (Fig. 1, Table 1, *SI Appendix*, Fig. S1, and Datasets S1–S9). A combination of HL and HS is common to field-grown plants during midday at temperate and tropical regions worldwide and was shown to have an adverse impact on photosynthesis, plant growth, and plant survival compared with each of its different components (HL or HS) applied individually (19–22). Applying a combination of the two components to the same leaf (HL+HS) resulted in a local response that included transcripts specific to light or heat stress, as well as transcripts unique to the stress combination (Fig. 1*A*). This state of stress combination generated a systemic signal(s) that resulted in a systemic response to stress combination in systemic leaves (Fig. 1*A*). Although the systemic response of plants to a local state of stress combination (HL+HS) included transcripts unique to each individual stress, as well as to the stress combination, its composition was nonetheless different from that of local leaves, representing an overall overlap of ∼25 to 30% with just 51 transcripts common between the stress combination-unique transcripts of local and systemic leaves (Fig. 1 *B* and *C*). Furthermore, although the response of local and systemic leaves of plants individually and locally subjected to HL or HS included many hydrogen peroxide (H_2_O_2_)-, HL-, HS-, and salicylic acid (SA)-response transcripts, these transcripts were not highly represented in the systemic response of plants simultaneously subjected to HL+HS (Table 1). These results demonstrate that plants are capable of integrating two different systemic signals (one for heat and one for high light), that the response of plants to stress combination in systemic leaves differs from that of local leaves, and that compared with the systemic response of plants to HL or HS individually applied to a single leaf, the systemic response of plants to the two stresses applied simultaneously to the same leaf (HL+HS) is less comprehensive, at least when it comes to HL-, HS-, SA-, or H_2_O_2_-response transcripts (Fig. 1 and Table 1).

**Table 1.** Presence of hormone-, ROS-, HL-, and HS-response transcripts in the different groups of transcripts significantly up-regulated in local and systemic leaves of plants subjected to HL and/or HS simultaneously applied to the same or two different leaves of the same plant

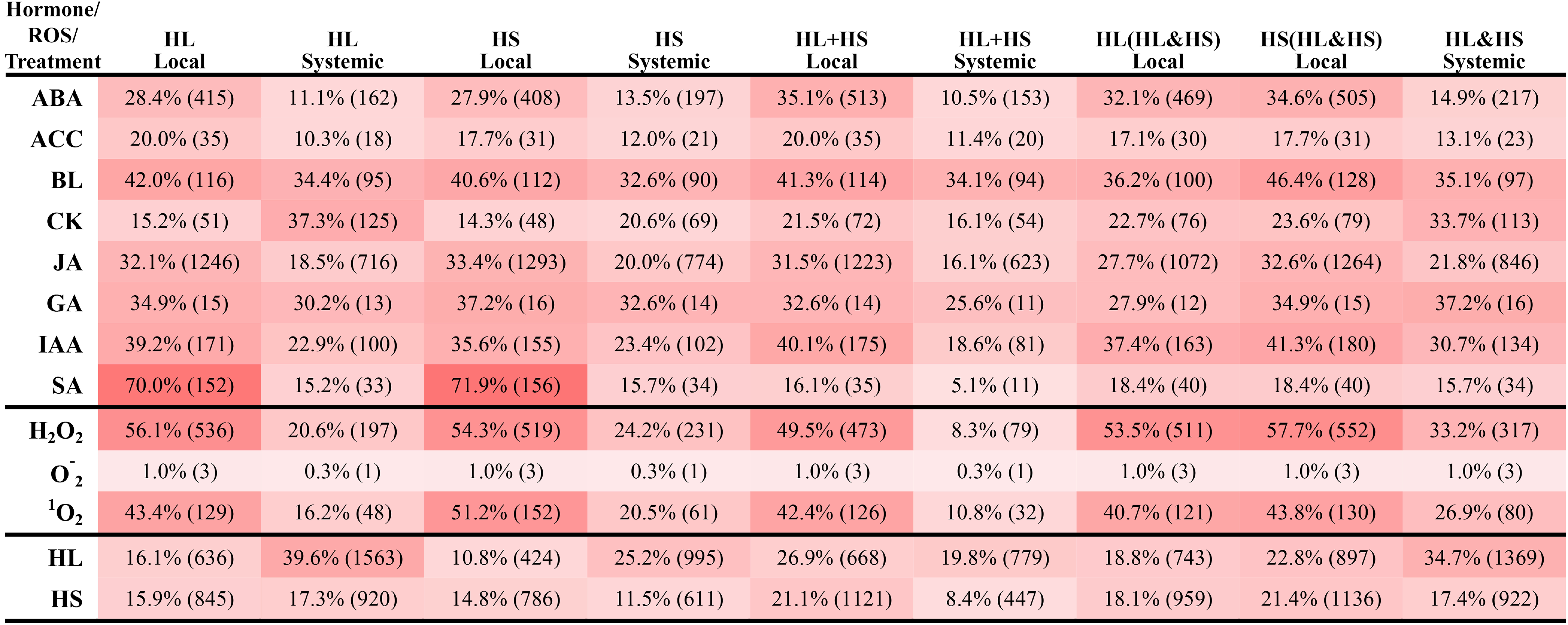

ABA, abscisic acid; ACC, 1‐aminocyclopropane‐1‐carboxylic acid; BL, brassinolide; CK, cytokinin; GA, gibberellin; IAA, auxin. All transcripts included were significantly different from controls at *P* < 0.05 (negative binomial Wald test followed by a Benjamini–Hochberg correction). Shading intensity is proportional to percent representation level of each hormone-, ROS-, or treatment-specific transcripts in the different tissues.

### Systemic Response of Plants to a Combination of High Light and Heat Stress Applied to Two Different Leaves of the Same Plant.

**Fig. 2.**
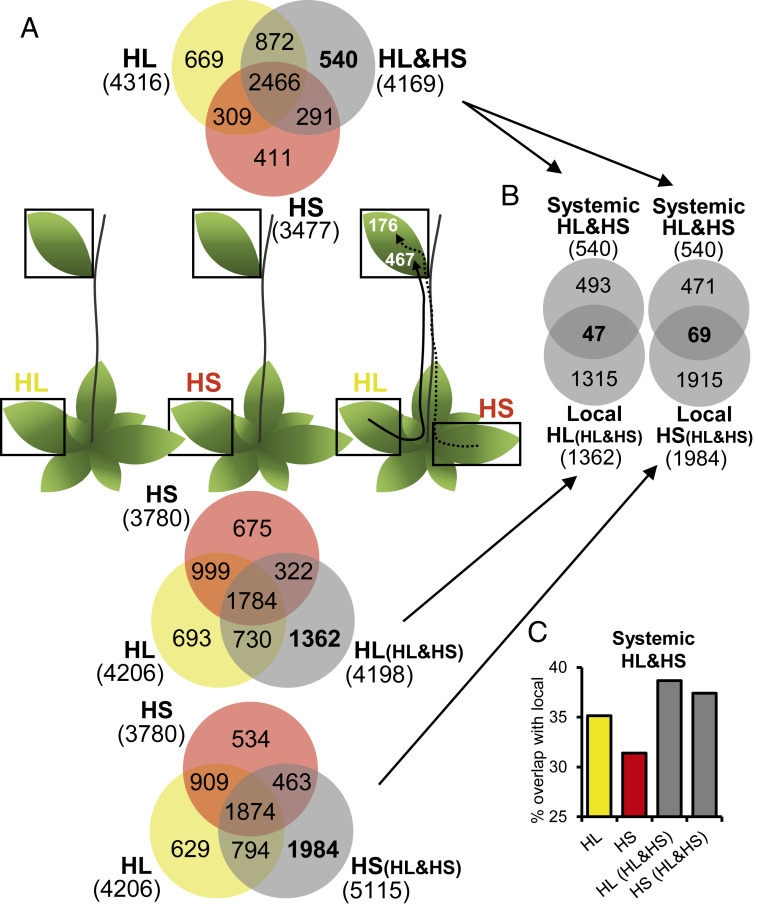
Plants can integrate two different systemic signals generated simultaneously at two different leaves of the same plant during stress combination. (*A*) Overlap between the systemic transcriptomic response of plants to a local application of HL, HS, or a combination of heat and light stress simultaneously applied to two different leaves (HL&HS). Venn diagrams for the overlap between the different responses are shown at the bottom for local leaves and at the top for systemic leaves. Black arrows in HL&HS plants represent the number of HL-specific (solid), or HS-specific (dashed) transcripts common between local and systemic leaves. (*B*) Venn diagrams showing the overlap between stress combination-specific transcripts from local (HL or HS) and systemic leaves. (*C*) Bar graph showing the percent overlap between the systemic HL&HS response and local HL, HS, HL(HL&HS), or HS(HL&HS) responses. All experiments were repeated at least three times with 40 plants per biological repeat. All transcripts included in the figure were significantly different from controls at *P* < 0.05 (negative binomial Wald test followed by Benjamini–Hochberg correction). HL(HL&HS) and HS(HL&HS) denote a local HL- or HS- treated leaf of a plant subjected to HS or HL on another local leaf, respectively.

We next examined the systemic response of plants to a combination of heat and light stress applied to two different leaves (Fig. 2, Table 1, and Datasets S1–S9). Applying a combination of light and heat stress to two different leaves of the same plant (HL&HS) resulted in local responses in the two different leaves that included transcripts specific to light or heat stress, as well as transcripts unique to the stress combination (Fig. 2*A*). This state of stress combination generated at least two different systemic signals that resulted in a systemic response to stress combination in systemic leaves (Fig. 2*A*). Although the systemic response of plants to a local state of stress combination (HL&HS) included transcripts unique to each individual stress, as well as to the stress combination, its composition also differed from that of local leaves, representing an overall overlap of ∼30 to 35%, with only 47 or 69 transcripts common between the stress combination-unique transcripts of local and systemic leaves (Figs. 1 and 2*B*). Interestingly, compared with the systemic response of plants to two different stresses applied to the same leaf (HL+HS), the response of systemic leaves of plants subjected to two different stresses, each applied to a different leaf (HL&HS), included a higher proportion of H_2_O_2_-, HL-, HS-, and SA-response transcripts (Table 1). These results demonstrate that plants are capable of integrating two different systemic signals (one for heat and one for high light), generated at two different leaves, that the response of plants to stress combination in systemic leaves is different from that of local leaves, and that the systemic response of plants to two different stresses simultaneously applied to two different local leaves (HL&HS) could potentially be different and more comprehensive compared with the systemic response of plants to two different stresses simultaneously applied to the same leaf (HL+HS) (Figs. 1 and 2 and Table 1).

### Comparing the Systemic Response of Plants to Stress Combination Applied to the Same or Two Different Leaves.

**Fig. 3.**
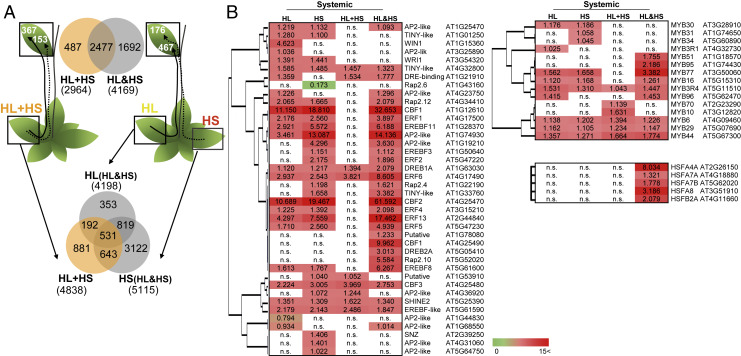
The systemic response of plants to two different stresses simultaneously applied to two different local leaves is more extensive than the response to two different stresses simultaneously applied to the same local leaf. (*A*) Overlap between the systemic transcriptomic responses of plants to a combination of heat and light stress applied to the same (HL+HS) or different (HL&HS) local leaves. Venn diagrams for the overlap between the different responses are shown at the bottom for local leaves and at the top for systemic leaves. Black arrows represent the number of HL-specific (solid) or HS-specific (dashed) transcripts common between local and systemic leaves. (*B*) Heat maps showing the expression pattern of TFs belonging to the ethylene response (AP2-EREBPs), MYB, and heat shock factor (HSF) families (24) in systemic tissues of plants subjected to a local treatment of HL, HS, HL+HS, or HL&HS. All transcriptomics experiments were repeated at least three times with 40 plants per biological repeat (*P* < 0.05, negative binomial Wald test followed by Benjamini–Hochberg correction).

The results presented in Figs. 1 and 2 and Table 1 suggest that the systemic response of plants to abiotic stress combination applied to the same (HL+HS) or two different (HL&HS) leaves could be different. Therefore, we conducted a more extensive analysis of the differences between these two systemic responses. As shown in Fig. 3*A*, the systemic response of plants to two different stresses applied simultaneously to two different leaves (HL&HS) was different and more extensive than the response to two different stresses applied to the same leaf (HL+HS). Although both systemic responses included transcripts unique to light or heat stress, as well as unique to the stress combination, the systemic response to two different stresses applied to two different leaves (HL&HS) was more comprehensive, with ∼1,200 additional transcripts (Fig. 3*A*). In addition, although an overlap of 2,477 transcripts was found between the two systemic responses (HL&HS and HL+HS) (Fig. 3), there were many more systemic transcripts unique to the state of two different stresses applied to two different leaves (HL&HS stress combination: 1,692 [Fig. 3*A*], as well as 540 transcripts unique to the stress combination [Fig. 2*A*]), compared with that of two different stresses applied to the same leaf (HL+HS stress combination: 487 [Fig. 3*A*], with only 276 transcripts unique to stress combination [Fig. 1*A*]), with a higher percent overlap between local and systemic responses under conditions of HL&HS (Figs. 1*C* and 2*C*).

In addition, compared with the systemic response of plants to two different stresses simultaneously applied to the same leaf (HL+HS), the representation of many hormone-, HL-, HS-, and ROS-response transcripts was higher in the systemic response of plants to two different stresses simultaneously applied to two different leaves (HL&HS) (Table 1). Analysis of different groups of transcription factors (TFs) involved in the integration of different abiotic stress-specific signals during stress combination (24) further revealed that, in contrast to systemic leaves of plants subjected to a local treatment of HL, HS, or HL and HS simultaneously applied to two different leaves (HL&HS), many of these TFs were not expressed in systemic leaves of plants subjected to a local treatment of two different stresses simultaneously applied to the same leaf (HL+HS) (Fig. 3*B*). These included, for example, Rap2.12, CBF1, ERF1, EREBF11, MYB16, and MYB77. Therefore, the systemic response of plants to two different stresses simultaneously applied to two different leaves is different and more extensive than the response to two different stresses simultaneously applied to the same leaf (Figs. 1–3 and Table 1).

### Leaf-To-Leaf Communication in Plants Subjected to Stress Combination Applied to Two Different Leaves.

**Fig. 4.**
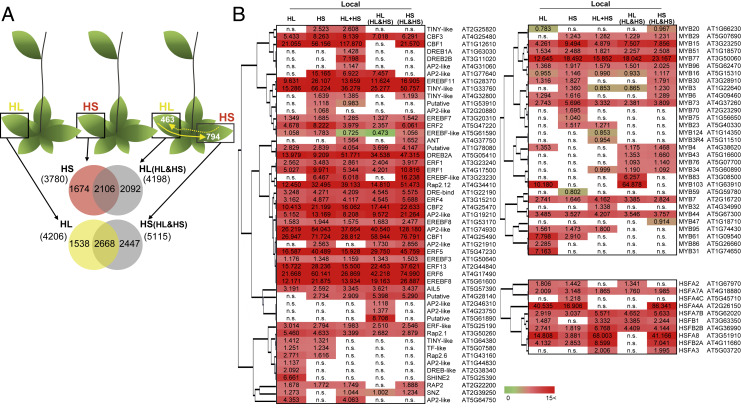
Overlap between the local transcriptomic responses of two different leaves of the same plant, one subjected to HL and the other to HS simultaneously. (*A*) Venn diagrams for the overlap between the different local responses of plants subjected to HL, HS, or HL&HS. Yellow arrows represent the number of HL-specific (solid) or HS-specific (dashed) transcripts common between the two different local leaves. (*B*) Heat maps showing the expression pattern of TFs belonging to the AP2-EREBP, MYB, and HSF families (24) in local tissues of plants subjected to HL, HS, HL+HS, or HL&HS. All transcriptomics experiments were repeated at least three times with 40 plants per biological repeat (*P* < 0.05, negative binomial Wald test followed by Benjamini–Hochberg correction).

The differences observed between the response of plants to two different stresses applied to the same or two different leaves (Figs. 1–3 and Table 1) could result from the two different local leaves subjected to the two different stresses exchanging signals with each other (3, 15, 23). Indeed, heat-specific transcripts could be found in local leaves subjected to light stress- and light-specific transcripts could be found in local leaves subjected to heat stress in plants simultaneously subjected to light and heat stress on two different leaves (HL&HS; Fig. 4*A*). In addition, as shown in Fig. 4*B*, comparing the expression pattern of TFs involved in the integration of different abiotic stress-specific signals during stress combination (24) between local leaves subjected to HL, HS, HL(HL&HS), and HS(HL&HS) reveals that several HL-response TFs are expressed in local HS(HL&HS) leaves (e.g., SNZ, MYB4), and several-HS response TFs are expressed in local HL(HL&HS) leaves (e.g., At1g21910, At4g28140, MYB29). These findings, as well as the overlap in overall heat and light stress responses of the two different local leaves (Fig. 2) and the similarity in hormone- and ROS-response transcript representation between the two different local leaves [HL(HL&HS) and HS(HL&HS); Table 1], demonstrate that in addition to generating two different systemic signals that integrate and travel to remote systemic tissues (Fig. 3), the two local leaves subjected to two different stresses can communicate with each other using stress-specific systemic signals (Fig. 4), affecting gene expression patterns in each other.

### SAA to Light or Heat Stress in Plants Subjected to Abiotic Stress Combination Applied to the Same or Two Different Leaves.

**Fig. 5.**
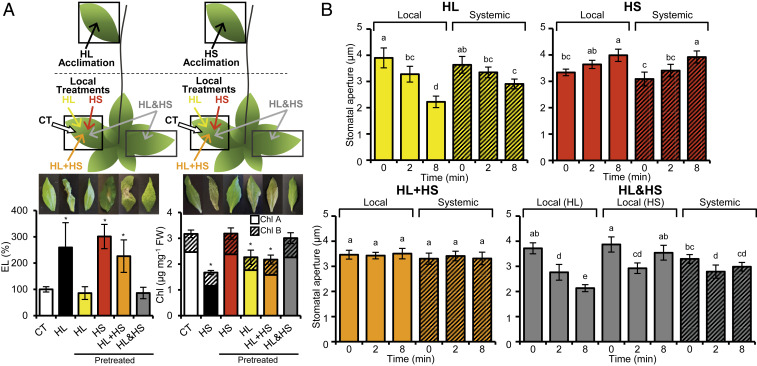
Acclimation of systemic leaves and stomatal aperture responses to stress combination. (*A*, *Top*) Schematic representation of how experiments were conducted. Local leaves were subjected to a short (15 min) pretreatment of HL, HS, HL+HS, or HL&HS, and plants were allowed to acclimate for 45 min. Following acclimation, systemic leaves were challenged with damaging levels of HL or HS, sampled, photographed and subjected to tissue injury assays (ion leakage for HL and chlorophyll content for HS). (*A*, *Middle*) Representative systemic leaf images. (*A*, *Bottom*) Measurements of systemic leaf injury: increase in ion leakage for HL (*Left*) and decrease in chlorophyll content for HS (*Right*). All acclimation experiments were repeated at least three times with 10 plants per biological repeat. Data are mean ± SD. *P < 0.05, two-way ANOVA followed by Tukey’s post hoc test. CT, control; HL or HS, control plants subjected to a systemic HL or HS stress treatment without pretreatment, respectively; Pretreated, plants in which a local leaf was subjected to HL, HS, HL+HS, or HL&HS treatment before the systemic HL or HS treatment; EL, electrolyte leakage. (*B*) Stomatal aperture in local and systemic leaves of plants subjected to a local HL, HS, HL+HS, or HL&HS treatment. All experiments were repeated at least three times with 500 stomata per plant and 10 plants per biological repeat. Data are presented as mean ± SD. Different letters denote significance at *P* < 0.05 (ANOVA followed by a Tukey’s post hoc test).

Enhanced expression of stress-response transcripts in systemic leaves could lead to enhanced plant acclimation (i.e., SAA) (5–7, 15). The differences in transcript expression observed between systemic tissues of plants integrating two different systemic signals originating from the same or different leaves (Figs. 1–4 and Table 1) prompted us to compare their SAA to light or heat stress. For this purpose, we subjected local leaves to a short pretreatment of HL, HS, HL+HS, or HL&HS; allowed plants to acclimate; and challenged the systemic leaves with damaging levels of HL or HS (5, 6, 15). Strikingly, although acclimation of systemic tissues to light (evident by a decrease in ion leakage upon acclimation) or heat (evidenced by the maintenance of high chlorophyll content upon acclimation) was observed when the different stresses were applied to local leaves individually (HL or HS) or simultaneously to two different leaves (HL&HS), when the two different stresses were simultaneously applied to the same leaf (HL+HS), no SAA to light or heat stress was observed (Fig. 5*A*). This finding demonstrates that the outcome of integrating two different systemic signals generated at the same or different leaves is different not only in transcript expression (Figs. 1–4 and Table 1), but also in plant acclimation (Fig. 5*A*).

### Regulation of Stomatal Aperture during Stress Combination.

Because transcript expression and plant acclimation are associated with stomatal responses that could also be coordinated between different leaves and required for plant acclimation (3, 15, 23, 25), and because stomatal responses to light or heat stress are opposing (opening during heat stress and closing during intense light stress) (20), we measured changes in stomatal aperture in local and systemic leaves of plants subjected to the two different stress combinations: HL&HS and HL+HS. As expected, local leaves subjected to light stress displayed stomatal closure, while local leaves subjected to heat stress displayed stomatal opening (Fig. 5*B*) (15, 22, 23). Interestingly, stomata of local leaves subjected to light stress while the other leaf was subjected to heat [HL(HL&HS)] closed, while stomata of local leaves subjected to heat stress while the other leaf was subjected to light stress [HS(HL&HS)] closed and then opened (Fig. 5*B*). A similar closing and opening response was observed in systemic leaves of plants subjected to the two different stresses applied to two different leaves (systemic HL&HS). The closing and then opening response of stomata in systemic (HL&HL) or local leaves of plants subjected to a combination of HL&HS [HS(HL&HS)] suggest that the systemic response to HL (closing) could be faster than the systemic response to HS (opening), but the systemic response to HS overcomes the systemic response to HL (Fig. 5*B*). In contrast, stomatal responses were dampened, with no significant opening or closing responses in local and systemic leaves of plants subjected to the two different stresses applied simultaneously to the same leaf (local or systemic leaves of HL+HS) (Fig. 5*B*).

### Activation of the ROS Wave during Stress Combination.

**Fig. 6.**
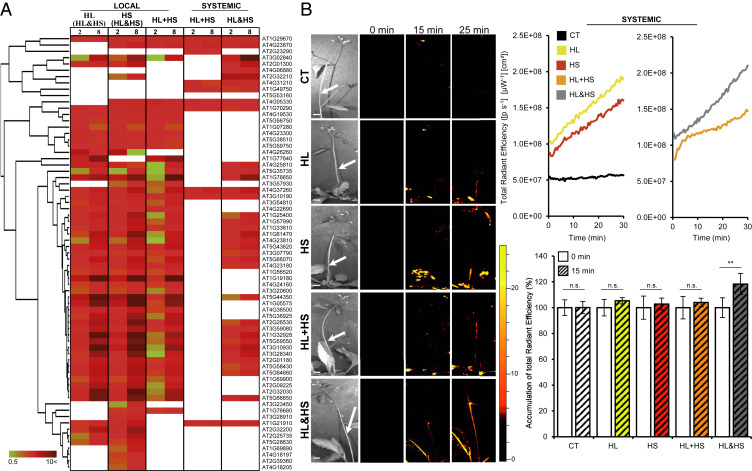
Systemic accumulation of ROS in mature bolting plants simultaneously subjected to two different stresses. (*A*) Heat maps showing the expression of transcripts associated with systemic ROS accumulation (6) in local leaves of plants subjected to light or heat stress while the other leaf is simultaneously subjected to the other stress [HL(HL&HS) or HS(HL&HS), respectively] or HL and HS applied to the same leaf (HL+HS), and in systemic leaves of plants subjected to a local treatment of HL and HS applied to the same (HL+HS) or two different (HL&HS) leaves (*SI Appendix*, Fig. S2). Transcripts included in *A* and in *SI Appendix*, Fig. S2 are significantly different from controls (*P* < 0.05; negative binomial Wald test followed by Benjamini–Hochberg correction). (*B*) Representative images (*Left*), line graphs showing continuous in vivo ROS measurements (*Top Right*), and bar graphs showing measurements of ROS at 15 min after stress application (*Bottom Right*), at the middle of the inflorescence stem (corresponding to white arrows in images on the left). All experiments were repeated at least three times with five plants per biological repeat. Data are presented as mean ± SD, **P < 0.01, two-way ANOVA followed by Tukey’s post hoc test. CT, control; n.s., no significant differences with respect to control. (Scale bars, 1 cm.)

One of the central systemic signaling pathways required for SAA to occur in plants is the ROS wave, an autopropagating cell-to-cell process of ROS production that accompanies the systemic signal (3, 5–8, 13–15). Comparing the pattern of ROS wave-associated transcripts (6) between local and systemic tissues of plants subjected to two different stresses applied to the same or two different leaves revealed that although many ROS wave-associated transcripts were up-regulated in the local tissues of plants subjected to two different stresses applied to the same or different leaves (i.e., local HL of HL&HS, local HS of HL&HS, and local HL+HS), as well as in systemic tissues of plants subjected to two different stresses applied simultaneously to two different leaves (i.e., systemic HL&HS), few ROS wave-associated transcripts were up-regulated in systemic tissues of plants simultaneously subjected to light and heat stress applied to the same leaf (systemic HL+HS) (Fig. 6*A* and *SI Appendix*, Fig. S2). This observation was supported by the abundance of H_2_O_2_- and ^1^O_2_-response transcripts in systemic tissues of HL&HS plants compared with systemic tissues of HL+HS plants (Table 1), as well as by in vivo measurements of ROS accumulation (8) in systemic tissues of similar size and age plants containing an inflorescence stem subjected to heat, light, and heat and light combinations applied to the same or different leaves (Fig. 6*B*). Although heat and/or light stresses applied individually or in combination to the same or to two different local leaves of plants (at the two different developmental stages) triggered the ROS wave, compared with all other treatments, the rate of ROS wave propagation was significantly faster when the two different stresses were simultaneously applied to two different leaves (HL&HS) (Fig. 6*B*, Table 1, and *SI Appendix*, Fig. S2).

### Accumulation of JA and SA in Local and Systemic Leaves during Stress Combination and Altered Systemic ROS Signaling during Stress Combination in the Allene Oxide Synthase (*aos*) Mutant.

**Fig. 7.**
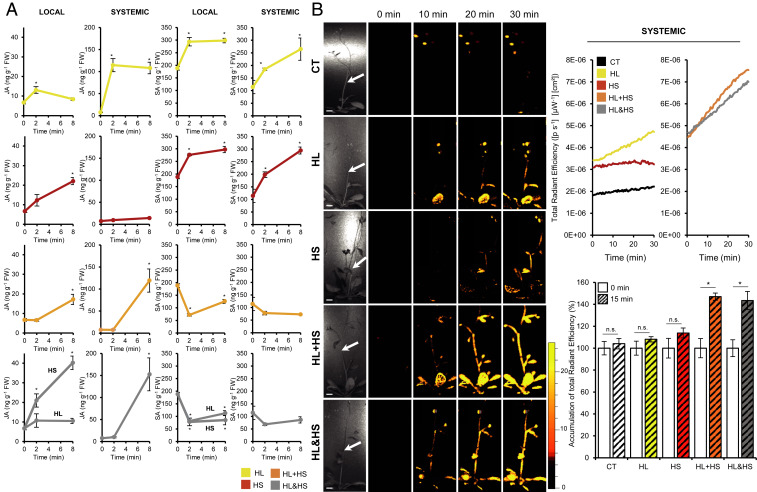
Quantitative analysis of JA and SA levels in wild-type plants and systemic accumulation of ROS in bolting *aos* mutants during stress combination. (*A*) Quantitative analysis of JA and SA in local and systemic wild-type leaves at 0, 2, and 8 min following the application of HL, HS, HL+HS, and HL&HS to local leaves. Hormone analysis was conducted using the same biological material used from transcriptomics analysis. Data are presented as mean ± SD. *P < 0.05, two-way ANOVA followed by Tukey’s post hoc test. (*B*) Representative images (*Left*), line graphs showing continuous in vivo ROS measurements (*Top Right*), and bar graphs showing measurements of ROS at 15 min after stress application (*Bottom Right*) at the middle of the inflorescence stem (corresponding to white arrows in images on *Left*) of *aos* mutant plants subjected to stress combinations. All experiments were repeated at least three times with five plants per biological repeat. Data are presented as mean ± SD. *P < 0.05, two-way ANOVA followed by Tukey’s post hoc test. CT, control; n.s., no significant differences with respect to control. (Scale bars, 1 cm.)

Because SA-regulated transcripts accumulated in local leaves subjected to HL or HS, but not in local leaves of HL+HS plants (Table 1), and because JA was found to play a key role in the protection of plants subjected to a combination of HL and HS (22), we quantified the levels of SA and JA in local and systemic leaves of plants subjected to HL, HS, HL+HS, and HL&HS (Fig. 7*A*). In agreement with the hormone-response transcript expression results shown in Table 1, JA and SA accumulated in local leaves of plants subjected to HL or HS. The accumulation of JA was nevertheless delayed, while the accumulation of SA was completely suppressed in local leaves of HL+HS plants, which display a suppressed ROS wave initiation phenotype (Fig. 6), suggesting that rapid accumulation of these two hormones is needed to trigger the ROS wave in local leaves and induce SAA (Figs. 5*A* and 6). In contrast to plants subjected to HL+HS, local leaves of plants subjected to HL&HS accumulated JA at normal rates (Fig. 7*A*), and this accumulation could be involved in initiating the ROS wave in these plants (Fig. 6).

In contrast to JA, the accumulation of SA was completely blocked in local or systemic leaves in both HL+HS and HL&HS (Fig. 7*A*), suggesting that SA accumulation is suppressed in systemic leaves during stress combination. Interestingly, the suppression of local ROS wave initiation during HL+HS did not occur in the *aos* mutant, which is suppressed in JA accumulation (22), supporting a role for JA in suppressing the initiation of the ROS wave in local leaves of HL+HS plants (Fig. 6). The findings presented in Fig. 7 point to important roles for JA and SA in systemic signal integration during stress combination in plants.

### Systemic ROS Accumulation and Acclimation of Nonbolting Plants during Stress Combination.

**Fig. 8.**
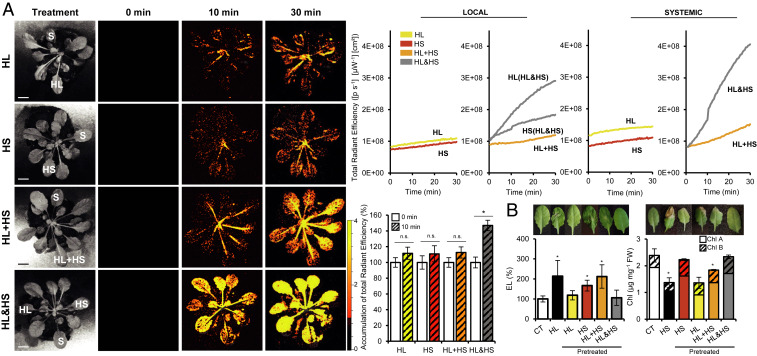
Systemic ROS accumulation and plant acclimation of nonbolting wild-type plants during stress combination. (*A*) Representative images (*Left*), line graphs showing continuous in vivo ROS measurements at different local and systemic leaves (*Top Right*; leaves monitored are indicated with letters on the left images), and bar graphs showing measurements of ROS at the indicated leaves at 10 min after stress application (*Bottom Right*). All experiments were repeated at least three times with five plants per biological repeat. Data are presented as mean ± SD. *P < 0.05, two-way ANOVA analysis followed by Tukey’s post hoc test. (*B*) Representative systemic leaf images (*Top*) and measurements of systemic leaf injury (increase in ion leakage for HL [*Left*] and decrease in chlorophyll content for HS [*Right*]) (*Bottom*) of nonbolting plants subjected to stress combination. All acclimation experiments were repeated at least three times with 10 plants per biological repeat. Data are presented as mean ± SD. *P < 0.05, two-way ANOVA followed by Tukey’s post hoc test. CT, control; HL or HS, control plants subjected to a systemic HL or HS stress treatments without pretreatment, respectively; Pretreated, plants in which a local leaf was subjected to a HL, HS, HL+HS or HL&HS treatment before the systemic HL or HS treatment; EL, electrolyte leakage; n.s., no significant differences with respect to control; S, systemic tissue. (Scale bars, 1 cm.)

Because the inability of local leaves of HL+HS plants to rapidly induce the ROS wave (Fig. 6) could also result from their metabolic and hormonal state at the reproductive stage, we tested whether a similar integration of systemic ROS signals (Fig. 6) and plant acclimation (Fig. 5*A*) occurs in young vegetative-stage plants. As shown in Fig. 8*A*, the systemic ROS wave response of young, nonbolting plants to two different stresses simultaneously applied to the same leaf (HL+HS) was stronger than that of reproductive-stage bolting plants (HL+HS) (Fig. 6), compared in each developmental stage with the two different stresses individually applied to a single leaf (HL or HS). This finding supports the possibility that differences in metabolic state, physiological activity, and/or hormone levels are affecting the integration of systemic signals and the triggering of the ROS wave by local leaves subjected to a combination of HL and HS (HL+HS). Nevertheless, even in young vegetative-stage plants, the systemic ROS wave response to two different stresses simultaneously applied to two different leaves (HL&HS) was stronger than that to two different stresses simultaneously applied to the same leaf (HL+HS) (Figs. 6 and 8*A*). Similarly, plant acclimation to stress combination also primarily occurred when the two different stresses were applied to two different leaves of young vegetative-stage or bolting plants (HL&HS) (Figs. 5*A* and 8*B*).

## Discussion

**Fig. 9.**
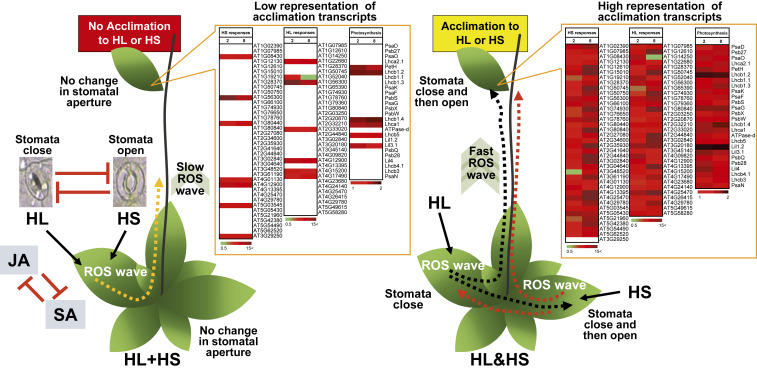
Model showing that the manner in which plants sense the different stresses that trigger systemic signals during stress combination (i.e., at the same or different leaves) makes a significant difference in how fast and efficient systemic ROS signals and transcriptomic, hormonal, stomatal, and acclimation responses are triggered. When two different stresses are simultaneously applied to the same leaf (HL+HS), the systemic response is suppressed. This is reflected in the expression of systemic HL- or HS-response and photosynthetic-associated transcripts (boxed heat maps; *SI Appendix*, Fig. S3 and Table 1), rates of SA and JA accumulation (Fig. 7*A*), accumulation of systemic ROS (dashed orange arrow; Figs. 6 and 8*A*), and the lack of SAA of systemic leaves (Figs. 5*A* and 8*B*). In contrast, when the two different stresses are applied to two different leaves of the same plant, the rate of systemic ROS accumulation is faster (dashed black and red arrows; Figs. 6 and 8*A*); stomata display a rapid response (Fig. 5*B*); HL-, HS-, and photosynthetic-associated transcripts accumulate in systemic leaves (boxed heat maps; *SI Appendix*, Fig. S3 and Table 1); and SAA is induced (Figs. 5*A* and 8*B*). Plants are depicted as being able to integrate different systemic signals: however, the manner in which plants sense the different stresses that trigger these signals makes a significant difference in how fast and efficient they are able to acclimate.

To be effective in inducing plant acclimation (i.e., SAA; Figs. 5*A* and 8*B*), the systemic signal(s) generated at the local leaf (leaves) must trigger several different responses in systemic tissues. These include changes in transcript abundance (e.g., photosynthetic- and other light- or heat-specific transcripts; Fig. 9, Table 1, and *SI Appendix*, Fig. S3) (8–10), metabolite levels (5, 18), ROS accumulation (Figs. 6–8) (5, 8), and stomatal responses (Fig. 5*B*) (15, 22, 23). Moreover, changes in these parameters must be rapid and coordinated between the different parts of the plant (3, 15, 18, 23). Surprisingly, while different systemic signals generated individually (HL or HS) or simultaneously at two different leaves (HL&HS) induced acclimation to light or heat stress, two different stresses applied simultaneously to the same leaf (HL+HS) did not (Figs. 5*A* and 8*B*). This finding was supported by the lower representation of HL-, HS-, and many ROS- and hormone-response transcripts in systemic leaves of plants subjected to HL and HS simultaneously applied to the same leaf (HL+HS; Fig. 3*B* and Table 1); the lower representation of transcripts unique to the stress combination in the systemic leaves of plants subjected to HL and HS simultaneously applied to the same leaf (HL+HS; Figs. 1–3); and the suppressed stomatal and hormonal responses of local and systemic leaves of plants subjected to HL and HS simultaneously applied to the same leaf (HL+HS; Figs. 5*B* and 7), all compared with that of systemic leaves of plants subjected to the two different stresses simultaneously applied to two individual leaves (HL&HS; Figs. 1–3 and Table 1) (5–8).

One plausible mechanistic explanation for this observation is that the two different stresses applied to the same leaf (HL+HS) suppressed the initiation or reduced the intensity of the ROS wave (Figs. 6–9, Table 1, and *SI Appendix*, Fig. S2) that is absolutely required for SAA to these stresses (5, 6, 15). Another plausible explanation could be the opposing effects of the stress combination applied to the same leaf (HL+HS) on stomatal responses (Fig. 5*B*), which could also affect overall plant acclimation (10, 15, 23, 25). It is also possible that an interplay between JA and SA levels (Table 1 and Fig. 7*A*) is responsible (18). In support of this explanation is the finding that the suppression of ROS wave initiation during HL+HS is removed in a mutant deficient in JA accumulation (*aos*; Fig. 7*B*). Therefore, the rate at which JA accumulates at the local leaves of HL+HS plants (compared to HL&HS) and its potential effects on SA accumulation (Fig. 7*A*; also reflected in transcript accumulation in Table 1) could play an important role in how fast and efficient the single leaf subjected to HL+HS can initiate systemic signaling and SAA. Thus, when JA accumulation is suppressed in the *aos* mutant, this inhibition is removed, and systemic ROS responses can occur.

Further studies are needed to address the many intriguing questions related to this new and emerging field of systemic signal integration during abiotic stress in plants. As revealed by this work, plants can integrate different systemic signals, and they do so best when the two different signals originate from two different leaves (Fig. 9). Like more complex multicellular organisms (e.g., animals), plants can therefore integrate different systemic signals and improve their chances of survival within the complex and rapidly changing environment that occurs in nature.

## Materials and Methods

### Plant Material and Stress Treatments.

*A. thaliana* Col-0 (cv. Columbia-0) plants were grown in peat pellets (Jiffy-7; https://www.jiffygroup.com/) at 23 °C under long-day growth conditions (16-h light/8-h dark; 50 µmol m^−2^ s^−1^) for 40 to 55 d until the inflorescence stem measured 11 to 13 cm. Local leaves were exposed to HL, HS, HL+HS, or HL&HS (*SI Appendix*, Fig. S1 *A* and *B*). HL was applied by subjecting a single leaf to a light intensity of 1,700 µmol m^−2^ s^−1^ for 2 and 8 min using a ColdVision fiber optic LED light source (Schott; A20980). HS was induced by placing a heat block 2 cm underneath the treated leaf for 2 and 8 min, increasing the leaf temperature to 31 to 33 °C (*SI Appendix*, Fig. S1*C*) as described previously (23). The temperature of the treated and systemic leaves was continuously measured using an infrared camera (C2; FLIR Systems) (*SI Appendix*, Fig. S1*C*).

### RNA Sequencing.

Treated and untreated local and systemic tissues (*SI Appendix*, Fig. S1) were sampled at 0, 2, and 8 min following stress application; immediately frozen in liquid nitrogen; and used for RNA-seq analysis. Local and systemic tissues from 40 different plants were pooled for each biological repeat per time point and stress treatment, and the experiment was repeated in three different biological replicates. All experiments were conducted at the same time of day (9 AM to 12 PM), and all plants used for the experiments were of the same age and developmental stage. Total RNA was isolated and subjected to RNA sequencing and analysis as described previously (6). RNA library construction and sequencing were performed by the DNA Core Facility at the University of Missouri.

### Acclimation Assays, Stomatal Aperture, and In Vivo Measurements of ROS Accumulation.

SAA assays were performed as described previously (5, 6) with some modifications. One rosette leaf of each bolting plant was exposed to the different stresses as shown in *SI Appendix*, Fig. S1 for 15 min. Following the local stress treatments, plants were incubated for 45 min under controlled conditions. Following the recovery period, one cauline leaf located 7 to 11 cm above the rosette leaves in each plant was exposed to 1,700 µmol m^−2^ s^−1^ light using a fiber optic light source (Schott A20980) for 45 min for HL acclimation or dipped into a 42 °C (or 23 °C as a control) water bath for 60 min. The cauline leaves were then photographed and sampled for electrolyte leakage assay (HL acclimation) or for chlorophyll measurement (HS acclimation) immediately after HL or ay 6 d after HS (5, 6). Electrolyte leakage assays were performed as described previously (6). Chlorophyll measurements were performed as described previously (5). Acclimation assays were repeated at least three times with 10 plants per repeat. In vivo measurements of ROS accumulation were performed as described previously (8). Stomatal aperture measurements were performed as described previously (15, 23).

### Quantification of Hormone Levels.

Quantification of plant hormones was conducted using reverse-phase ultra-high-performance liquid chromatography (UHPLC) coupled with electrospray ionization tandem triple quadrupole mass spectrometry (ESI/TQ MSMS) using multiple reaction monitoring as described previously (26). In brief, *A. thaliana* leaves were flash-frozen in liquid nitrogen and ground to powder. Samples (70 mg) were shaken vigorously in isopropanol:H_2_O:HCl (2:1:0.002) for 1 h at 4 °C. Following extraction, dichloromethane was added, and samples were again shaken vigorously for an additional 30 min at 4 °C. Following centrifugation (3500 × *g* at 4 °C), the bottom layer was removed using a glass syringe. Samples were then dried under nitrogen, redissolved in 0.10 mL of MeOH and 1 mL of 1% acetic acid, purified over Oasis HLB columns (Waters), washed with 1% acetic acid, and eluted with 80% MeOH and 1% acetic acid. The eluted samples were then dried again under nitrogen and redissolved in 25 μL of MeOH and 25 μL of 1% acetic acid. A 10-μL volume of each sample was separated on a Waters Acquity UPLC BEH C18 column (2.1 × 150 mm; 1.7 μm) at 60 °C, using a Waters Acquity UHPLC system. A Waters Xevo TQ MSMS system was used to identify the different hormones, and absolute quantification was obtained using stable isotope label internal standards for JA and SA (26).

### Statistical Analysis.

Differentially expressed transcripts were defined as those that had a fold change with an adjusted *P* < 0.05 (negative binomial Wald test followed by Benjamini–Hochberg correction). Venn diagram overlaps were subjected to hypergeometric testing using the R package phyper. Acclimation and stomatal aperture measurement data are presented as mean ± SD. Statistical analysis was performed by two-way ANOVA followed by Tukey’s post hoc test (*P < 0.05; **P < 0.01). Different letters in stomatal measurements denote statistical significance at *P* < 0.05.

### Data Availability.

Data supporting the findings of this work are provided in the main paper and *SI Appendix*. Raw and processed RNA-seq data files were deposited in the GEO database (https:// www.ncbi.nlm.nih.gov/geo/) (accession no. GSE138196).

## Supplementary Material

Supplementary File

Supplementary File

Supplementary File

Supplementary File

Supplementary File

Supplementary File

Supplementary File

Supplementary File

Supplementary File

Supplementary File
