# Supplementary material for: Systemic signaling during abiotic stress combination in plants
Source: Proc Natl Acad Sci U S A. 2020 May 29;117(24):13810–20. doi: 10.1073/pnas.2005077117 (PMC7306788; doi:10.1073/pnas.2005077117)
Supplement: Supplementary File [file pnas.2005077117.sapp.pdf]

PNAS
www.pnas.org

Supplementary Information for

Systemic signaling during abiotic stress combination in plants

Sara I. Zandalinas[1], Yosef Fichman[1], Amith R. Devireddy[1], Soham Sengupta[2], Rajeev K. Azad[2], and Ron Mittler[1,*]

*Corresponding author: Ron Mittler
Email: mittlerr@missouri.edu

This PDF file includes:

Legends for Datasets 1 to 9
Figures S1 to S3
Table S1

Legends for Datasets 1 to 9

Data Set 1. Transcripts significantly upregulated ($p < 0.05$) in local leaves subjected to high light (HL) for 2 and 8 min.

Data Set 2. Transcripts significantly upregulated ($p < 0.05$) in local leaves subjected to heat stress (HS) for 2 and 8 min.

Data Set 3. Transcripts significantly upregulated ($p < 0.05$) in local leaves simultaneously subjected to high light and heat stress (HL+HS) for 2 and 8 min.

Data Set 4. Transcripts significantly upregulated ($p < 0.05$) in local leaves subjected to high light when heat stress is applied to another local leaf (HL – HL&HS) for 2 and 8 min.

Data Set 5. Transcripts significantly upregulated ($p < 0.05$) in local leaves subjected to heat stress when high light is applied to another local leaf (HS – HL&HS) for 2 and 8 min.

Data Set 6. Transcripts significantly upregulated ($p < 0.05$) in systemic leaves of plants subjected to a local treatment of high light (HL) for 2 and 8 min.

Data Set 7. Transcripts significantly upregulated ($p < 0.05$) in systemic leaves of plants subjected to a local treatment of heat stress (HS) for 2 and 8 min.

Data Set 8. Transcripts significantly upregulated ($p < 0.05$) in systemic leaves of plants subjected to a local treatment of high light and heat stress in the same leaf (HL+HS) for 2 and 8 min.

Data Set 9. Transcripts significantly upregulated ($p < 0.05$) in systemic leaves of plants subjected to a local treatment of high light and heat stress in two different leaves (HL&HS) for 2 and 8 min.

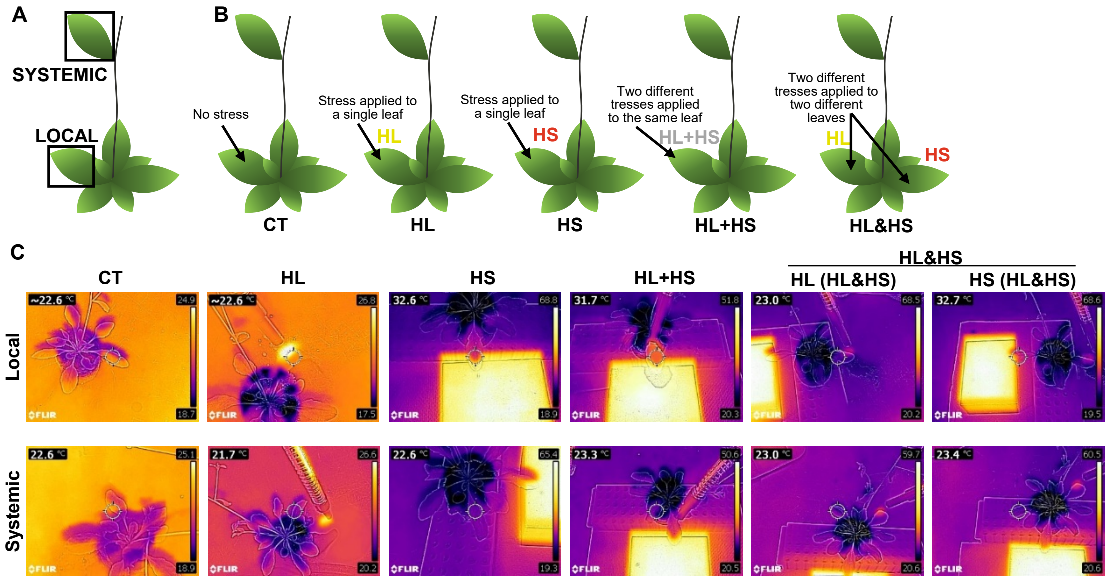

Figure S1. The experimental design used for local stress application. **(A)** Definition of local (treated) and systemic (non-treated) tissues of Arabidopsis plants. **(B)** The different local stresses applied. Local leaves were subjected to high light (HL, yellow; 1700 µmol m^{-2} s^{-1}), heat stress (HS, red; 31-33°C using a heat block) and high light and heat stress applied simultaneously to the same (HL+HS, orange) or two different (HL&HS) local leaves, for 2 and 8 min. No stress was applied for control (CT) plants. **(C)** FLIR camera images showing the temperature of treated (local, up) and systemic (bottom) leaves for each stress treatment (C2, FLIR systems AB).

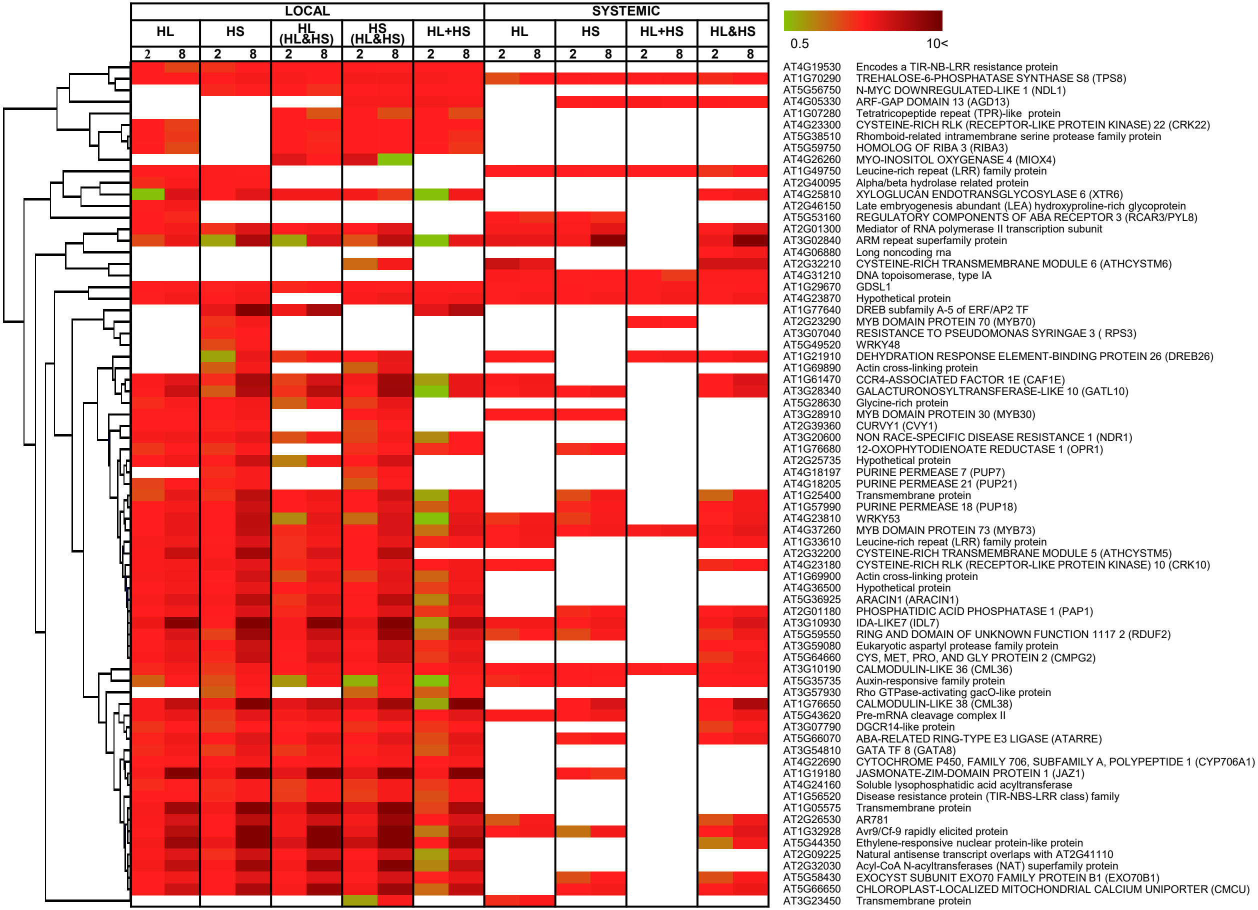

Figure S2. Complete heat maps for Figure 6A showing the expression of transcripts associated with systemic ROS accumulation in local and systemic leaves of plants subjected to a local treatment of light stress (HL), heat stress (HS), HL and HS applied to the same leaf (HL+HS), or HL and HS applied to two different leaves of the same plant (HL&HS).

A

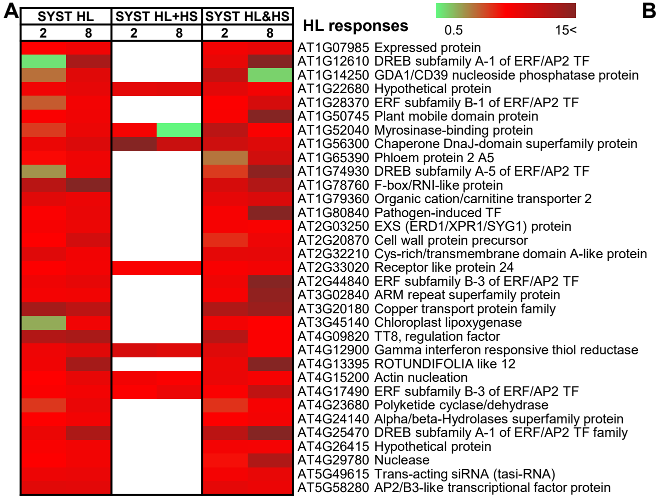

B

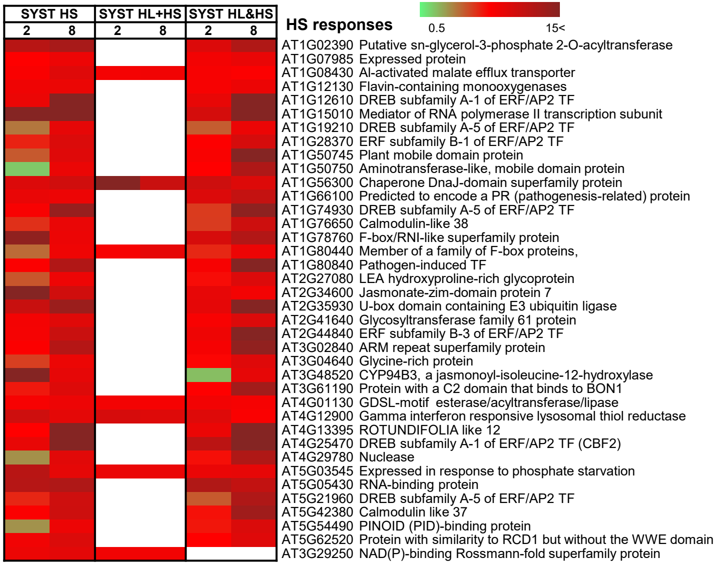

C

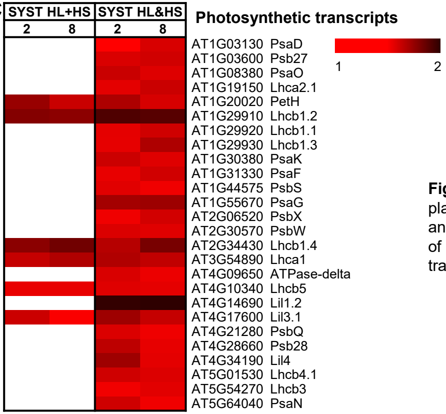

Figure S3. Expression of top HL- or HS- local response, and photosynthetic-associated transcripts in systemic leaves of plants subjected to a local treatment of light stress (HL), heat stress (HS), HL and HS applied to the same leaf (HL+HS), or HL and HS applied to two different leaves of the same plant (HL&HS) (from Figure 9). **(A-B)** Heat maps showing the accumulation of top HL- **(A)** or HS- **(B)** response transcripts. **(C)** Heat maps showing the accumulation of photosynthesis-associated transcripts. Photosynthetic-associated transcripts were obtained as described in (22).

	APX2 (AT3G09640)		APX1 (AT1G07890)		RbohD (AT5G47910)		RbohF (AT1G64060)		Zat10 (AT1G27730)		Zat12 (AT5G59820)	
	2	8	2	8	2	8	2	8	2	8	2	8
**Local HL**	2.91	4.78	1.13	1.43	0.92	1.25	1.14	1.04	2.40	14.92	1.12	5.54
**Systemic HL**	n.s.	n.s.	1.06	1.13	n.s.	n.s.	n.s.	n.s.	1.31	1.67	1.80	1.99
**Local HS**	3.99	6.05	1.26	1.15	0.95	1.88	1.14	1.10	1.11	24.40	1.09	6.40
**Systemic HS**	1.80	0.98	1.14	1.22	n.s.	n.s.	n.s.	n.s.	0.95	3.17	0.90	2.95
**Local HL+HS**	5.40	6.09	0.85	1.29	0.87	1.64	n.s.	n.s.	0.55	17.22	0.40	8.81
**Systemic HL+HS**	n.s.	n.s.	1.12	1.13	n.s.	n.s.	n.s.	n.s.	n.s.	n.s.	n.s.	n.s.
**Local HL(HL&HS)**	3.58	3.94	0.89	1.28	0.95	1.78	n.s.	n.s.	4.50	25.77	1.58	7.61
**Local HS(HL&HS)**	2.94	3.17	0.88	1.17	0.95	2.22	n.s.	n.s.	5.20	32.94	1.73	9.49
**Systemic HL&HS**	n.s.	n.s.	1.12	1.09	0.96	1.26	n.s.	n.s.	1.31	5.49	1.58	6.82

0 100

Table S1. Fold change in the expression of selected transcripts in local and systemic leaves of plants subjected to a loca ltreatment of HL, HS, HL+HS and HL&HS. Abbreviations: APX, ascorbate peroxidase; Rboh, respiratory oxidase burst homolog; Zat, Cys2/His2-type (C2H2) zinc finger protein.
